# Case Report: Cadonilimab plus anlotinib with radiotherapy for lung adenocarcinoma with pancreatic metastasis in later-line therapy

**DOI:** 10.3389/fimmu.2025.1610710

**Published:** 2025-07-18

**Authors:** Jianquan Yang, Zixin Li, Xuezhou Pang, Yakun Zhang, Beilei Zeng, Yan Gui, Daiyuan Ma

**Affiliations:** Department of Oncology, Affiliated Hospital of North Sichuan Medical College, Nanchong, Sichuan, China

**Keywords:** cadonilimab, anlotinib, lung adenocarcinoma, pancreatic metastasis, radiotherapy

## Abstract

Patients with advanced lung adenocarcinoma lacking driver gene mutations face the clinical dilemma of limited treatment options and poor prognosis in later-line therapy. Although immune checkpoint inhibitors (ICIs) combined with anti-angiogenic agents offer a promising approach, the optimal treatment strategy remains to be explored. In this report, we present a case of advanced lung adenocarcinoma with pancreatic metastasis treated with cadonilimab plus anlotinib and radiotherapy to the pancreatic lesion, resulting in 11 months of progression-free survival (PFS) and only minor side effects. This outcome suggests the potential value of cadonilimab and anlotinib in later-line therapy for advanced lung adenocarcinoma and provides a possible new treatment option for such patients.

## Introduction

Lung cancer is the malignant tumor with the highest incidence and mortality worldwide (1). Lung adenocarcinoma has become the most common histological subtype of non-small cell lung cancer (NSCLC) (2). Targeted therapy has significantly improved the prognosis of patients with driver gene-positive tumors (3, 4). However, a considerable proportion of these patients still experience poor outcomes after receiving first-line chemotherapy combined with immune checkpoint inhibitor (ICI) treatment. In recent years, based on the results of the IMpower-150 study, anti-angiogenic therapy combined with immunotherapy has offered a new treatment strategy for advanced lung adenocarcinoma patients without driver gene mutations (5). Cadonilimab, a novel PD-1/CTLA-4 bi-specific antibody, has shown excellent efficiency in solid tumors (6, 7). Furthermore, recent studies have shown that combining antiangiogenic drugs with ICIs can improve the prognosis of patients with mild side effects (8–10). In this study, we report a case of advanced lung adenocarcinoma in a patient who had received standard first-line treatment and could not tolerate second-line docetaxel chemotherapy,. The patient was subsequently treated with cadonilimab plus anlotinib in combination with radiotherapy for pancreatic metastases, resulting in 11 months of PFS with mild side effects. This “triple therapy” approach—immunotherapy, anti-angiogenic therapy, and local radiotherapy—may offer a novel treatment option for advanced pulmonary adenocarcinoma in later-line therapy.

## Case presentation

All procedures involving human participants complied with the ethical standards of the institutional and/or national research committee(s) and with the Declaration of Helsinki (as revised in 2013). Written informed consent was obtained from the patient.

**Figure 1:**
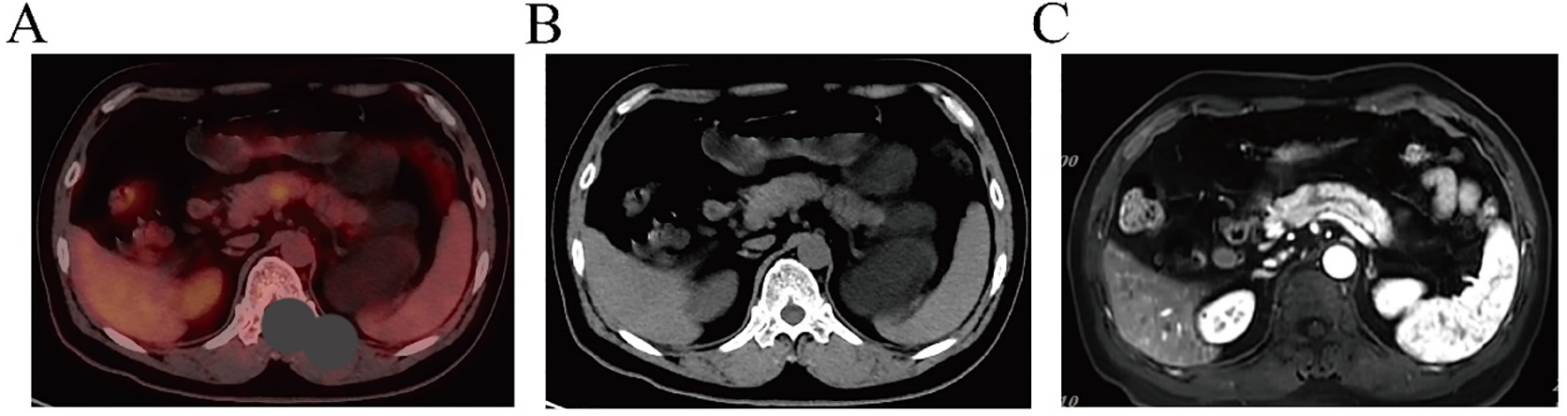
**(A, B)** PET/CT indicates pancreatic lesion as a metastatic tumor. **(C)** MRI features suggest that the pancreatic lesion is consistent with metastasis.

The patient was admitted to the hospital on December 14, 2023, for postoperative recurrence of lung adenocarcinoma and was receiving later-line therapy after first-line chemotherapy combined with immunotherapy. PET/CT revealed new metabolically active lesions in the pancreatic body (SUVmax = 5.2), (**Figure 1A, B**). MRI showed a lesion measuring approximately 1.3 cm × 1.1 cm in the pancreatic body by MRI (**Figure 1C**). The patient refused to undergo pancreatic puncture biopsy. Combined with the PET/CT and MRI results, the pancreatic lesion was considered a metastasis from lung cancer.

**Figure 2:**
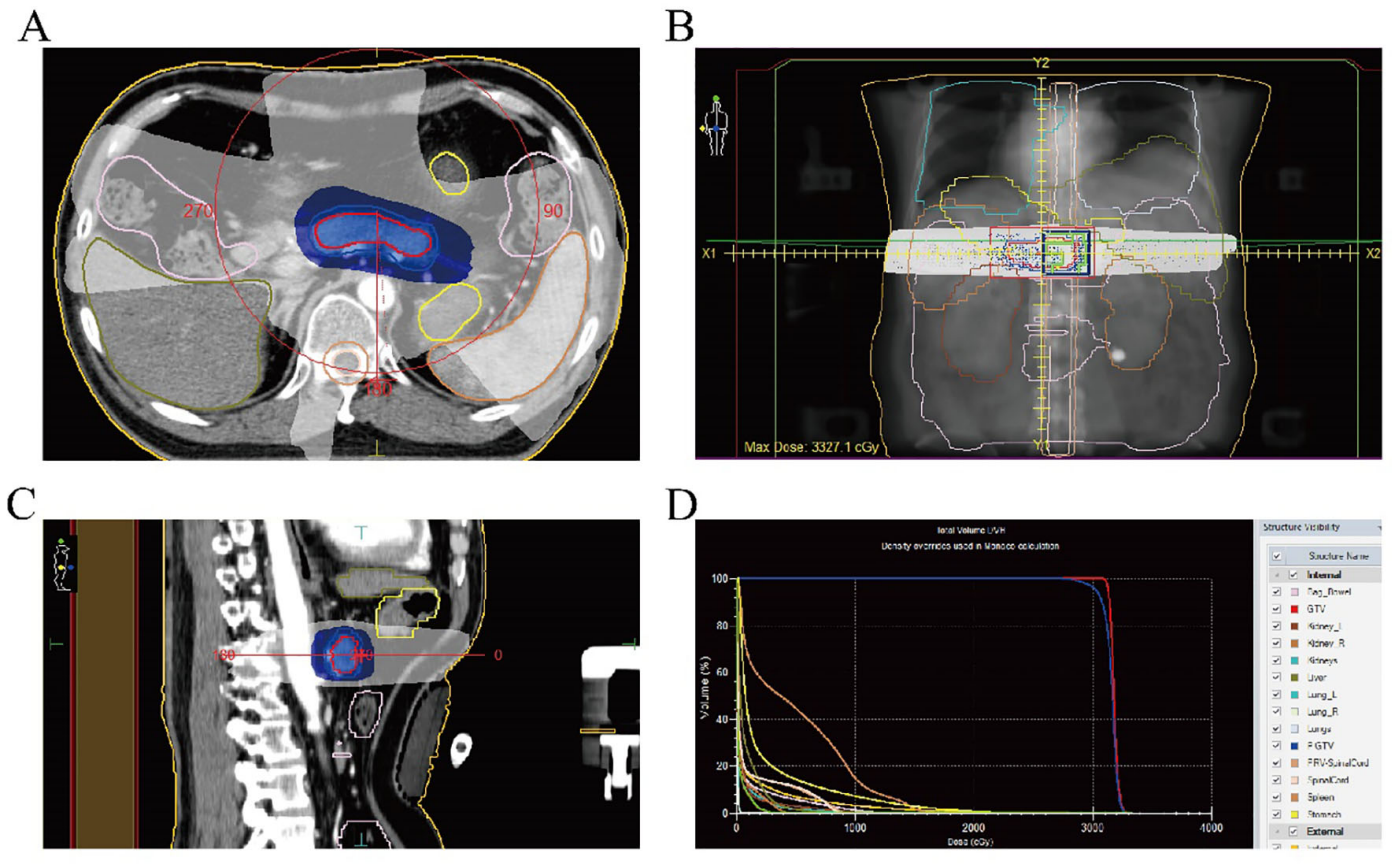
**(A)** Radiotherapy target area of the pancreatic metastasis (cross section); **(B)** Radiotherapy target of the pancreatic metastases (coronal view); **(C)** Radiotherapy target of the pancreatic metastases (sagittal view); **(D)** Dose-volume histogram of radiotherapy for the pancreatic metastasis.

This was considered a progressive disease after multiple lines of therapy in a driver gene-negative patient. Based on recent study results, the patient began treatment with cadonilimab (730mg, d1) plus (allotinib, 10mg D1-14, every 3 weeks) on January 23, 2024. Radiotherapy was also performed to the pancreatic metastasis at a dose of 45 Gy/15 Fx (**Figure 2**). CT scans performed after the second and fifth cycles of treatment showed stable disease (SD) in the pancreatic lesions. Elevated urinary microalbumin levels (1,810 mg) were observed after two cycles of treatment but returned to normal after 6 weeks of treatment with piperazine ferulate (50 mg, once daily). The patient continued receiving the cadonilimab plus anlotinib regimen.

**Figure 3:**
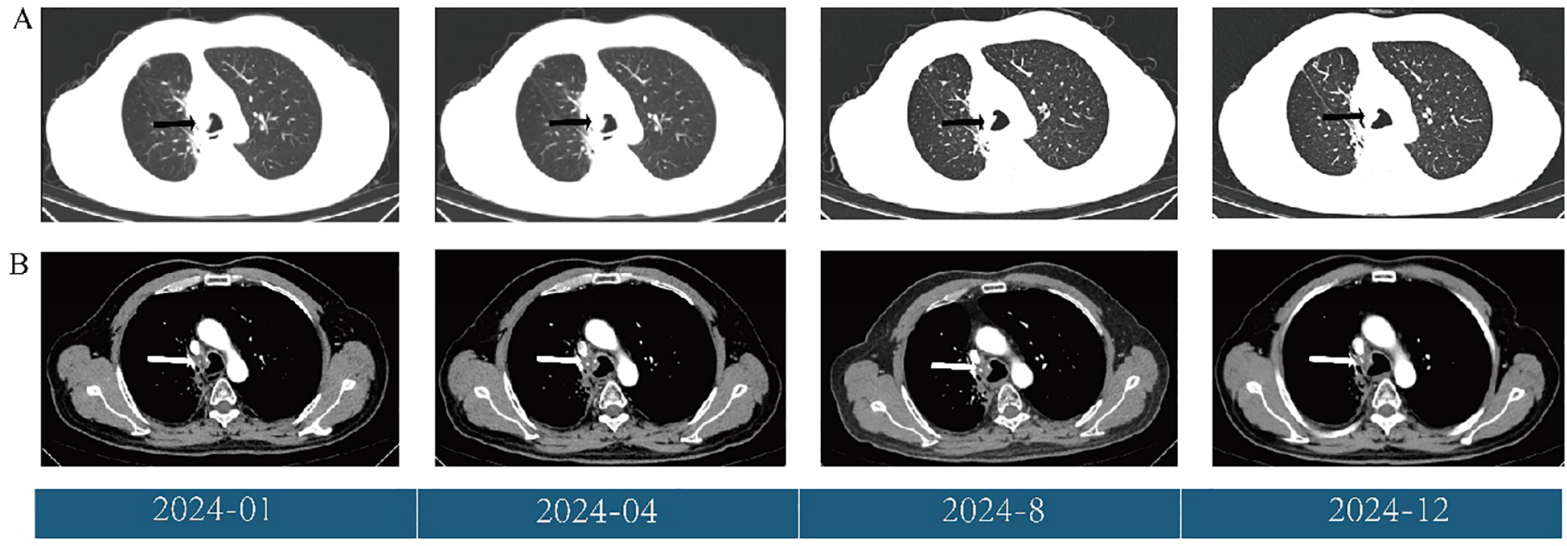
**(A)** Changes in the primary lung lesions (black arrow) during treatment, shown on CT (lung window). **(B)** Changes in the primary lung lesions (white arrow) during treatment, shown on CT (mediastinal window).

**Figure 4:**
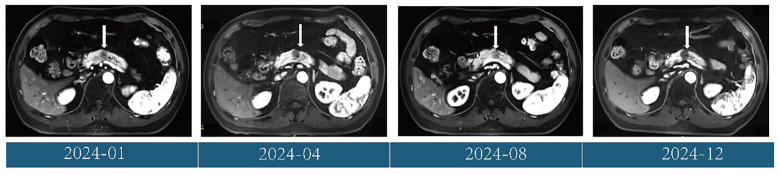
Changes in the pancreatic metastases (white arrow) during treatment, shown on MRI (T1 weighted image).

**Figure 5:**
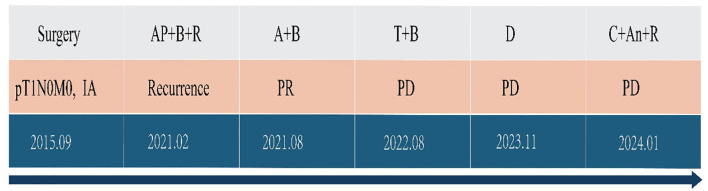
Antitumor treatment timeline. A, pemetrexed; P, cisplatin; B, bevacizumab; T, tislelizumab; C, cadonilimab; An, anlotinib; R, radiotherapy; PR, partial response; PD, progressive disease.

Follow-up CT scans were performed at the eighth and tenth cycles, both indicating stable disease. After 13 cycles, CT and MRI were performed on November 28, 2024, and the patient’s tumor response remained stable (**Figures 3**, **4**). According to the treatment timeline (**Figure 5**), the patient achieved 11 months of progression-free survival (PFS) with only minor side effects while receiving cadonilimab plus anlotinib and radiotherapy for pancreatic metastasis in later-line therapy.

## Discussion

Chemotherapy combined with ICIs remains the standard protocol for advanced NSCLC patients who are driver gene-negative and have low PD-L1 expression (tumor proportion score [TPS] < 5%). The KEYNOTE-189, IMpower150, and CameL studies have confirmed the survival advantage of this approach in non-squamous NSCLC (11, 12). However, in-depth analyses indicate that clinical benefits are limited in patients with low PD-L1 expression, and treatment-related toxicity—such as immune-related pneumonia, hepatitis, and chemotherapy-induced myelosuppression—is increased (13). Therefore, identifying more effective and less toxic treatment strategies has become a key focus of clinical research.

Compared with traditional PD-1+CTLA-4 monoclonal antibody combination therapies, cadonilimab can accurately target tumor-infiltrating lymphocytes due to its unique molecular structure. This improves efficacy while significantly reducing peripheral immunotoxicity (14). This advantage has been demonstrated in cervical cancer (7). However, its efficacy in other solid tumors, such as NSCLC, still requires validation through additional phase III clinical studies.

Previous studies have suggested that a “chemo-free” approach—combining anti-angiogenic therapy with immunotherapy —can improve outcomes in patients who are intolerant to chemoimmunotherapy. In this case, cadonilimab plus anlotinib was used for systemic treatment, with radiotherapy directed at pancreatic metastases. To date, the patient has achieved 11 months of PFS, which exceeds the 6-month PFS reported by Wang X et al. (15). The extended PFS in this case may be attributed to the addition of radiotherapy to the treatment plan.

As a rare metastatic site of lung adenocarcinoma, studies have shown that receiving local pancreatic radiotherapy can improve patients’ overall survival (OS). In this case, the patient received radiotherapy for pancreatic metastasis followed by systemic treatment and achieved a progression-free survival (PFS) of 11 months (16). Common side effects of pancreatic radiotherapy include gastrointestinal toxicity (such as ulcers and bleeding) and exocrine pancreatic insufficiency (17). In addition, if the radiation dose to the proximal duodenum or jejunum exceeds 50 Gy, long-term complications such as intestinal wall fibrosis, stenosis, or even obstruction may occur, with an incidence of 2–9%. Severe cases may require surgical intervention (18). In this case, the radiotherapy dose was 45 Gy, which not only achieved good control of pancreatic metastasis, but also showed no obvious gastrointestinal side effects and pancreatic exocrine function injury.

Although anlotinib has shown remarkable efficacy in the treatment of solid tumors such as NSCLC, its potential side effects require close attention. In this case, early signs of kidney injury were observed after two treatment cycles. Previous studies have shown that anlotinib can increase the risk of proteinuria in cancer patients (19). Proposed mechanisms include podocyte injury, hemodynamically mediated glomerular damage, and thrombotic microangiopathy (20–22). Therefore, early monitoring and effective management play a crucial role.

## Conclusion

We report a case of a patient with driver gene-negative primary lung adenocarcinoma who experienced disease progression following standard first- and second-line therapy, and subsequently achieved 11 months of PFS after treatment with cadonilimab plus anlotinib in combination with radiotherapy for pancreatic metastasis. This case highlights the potential value of a synergistic treatment regimen—immunotherapy + anti-angiogenesis + precision radiotherapy—for advanced driver gene-negative lung adenocarcinoma in later-line therapy, particularly in cases with pancreatic metastasis.

## Data Availability

The original contributions presented in the study are included in the article/Supplementary Material. Further inquiries can be directed to the corresponding authors.
